# Predictors of acute compartment syndrome of the lower leg in adults following tibial plateau fractures

**DOI:** 10.1186/s13018-021-02660-7

**Published:** 2021-08-16

**Authors:** Xiangtian Deng, Hongzhi Hu, Zhipeng Ye, Jian Zhu, Yiran Zhang, Yingze Zhang

**Affiliations:** 1 School of Medicine, Nankai University, Tianjin, 300071 People’s Republic of China; 2 Department of Orthopaedic Surgery of Hebei Province, The Third Hospital of Hebei Medical University, 139 Ziqiang Road, Shijiazhuang, 050051 Hebei People’s Republic of China; 3 NHC Key Laboratory of Intelligent Orthopeadic Equipment, The Third Hospital of Hebei Medical University, 139 Ziqiang Road, Shijiazhuang, 050051 Hebei People’s Republic of China; 4 Department of Orthopedics, Union Hospital of Tongji Medical College of Huazhong University of Science and Technology, Wuhan 430022 Wuhan, People’s Republic of China

**Keywords:** Acute compartment syndrome, Adults, Tibial plateau fractures, Prevalence rate, Predictors

## Abstract

**Background:**

Acute compartment syndrome (ACS) is an underestimated complication following tibial plateau fractures. Understanding predictors of ACS in the lower leg after a fracture of the tibial plateau may guide earlier diagnosis and promptly decompressed by surgical fasciotomy. To date, however, there are few large-scale sample literatures to investigate the predictors of it. The purpose of our study was to evaluate the prevalence rate of ACS associated with tibial plateau fractures and identified any such predictors with the development of ACS.

**Materials and methods:**

From January 2015 to January 2020, a total of 1119 consecutive patients (1119 fractures) including 703 males and 416 females with an average age of 40.7 years (18 to 80 years) in tibial plateau fractures who presented to a university-affiliated hospital with level-I trauma center were included. The presence of ACS and associated predictors were collected from patients’ electronic medical records. Associated predictors included gender, age, fracture pattern (open or closed), mechanisms of injury, fracture classification, and underlying disease. Univariate and multivariate logistical regression analyses were performed to identify the predictors of the development of ACS following tibial plateau fractures.

**Results:**

Of the 1119 fractures of the tibial plateau, 35 (3.1%) developed an acute compartment syndrome. On multivariate analysis, only younger patient age (odds ratio (OR) 2.57; 95% confidence interval (CI), 1.26 to 6.31; *P* = 0.003), and Schatzker VI type fracture (OR 5.78; 95% confidence interval (CI), 1.78 to 54.34; *P* = 0.021) were significantly associated with the development of ACS. Other variables did not reach statistical significance.

**Conclusion:**

Younger patient age and Schatzker VI type fracture were predictors of ACS of the lower leg in adults following tibial plateau fractures. Further studies in the prospective study are still needed to identify the potential risk factors associated with ACS in tibial plateau fractures.

## Introduction

Tibial plateau fractures are intra-articular fractures that account for approximately 1.6% of all fractures associated with adults in China [1], which are generally accepted that carry a risk of various complications. Generally, tibial plateau fractures are typically classified according to the Schatzker grading system, in which fractures are classified as types I–VI [2]. Acute compartment syndrome (ACS) is a clinical diagnosis that occurs within an enclosed myofascial compartment with an elevated intra-compartmental pressure which may result in an impairment of tissue perfusion, infection, loss of function, limb-threatening, amputation, and even death [3–5].

Among adults with tibial plateau fractures, the overall prevalence rate of subsequent ACS has been reported to range from 11 to 17% [6–9] and as high as 53% for medial knee fracture dislocations [10]. Although tibial plateau fractures are not commonly associated with ACS in adults, the knowledge of the prompt recognition, appropriate opportunity to emergency fasciotomy, and associated risk factors of ACS occurrence are important for orthopedic trauma surgeons. As the relatively lower prevalence rate of ACS in tibial plateau fractures, clinical diagnosis was challenging and difficult for clinicians [11], and ACS is an underestimated complication of tibial plateau fractures which could lead to devastating sequelae if not diagnosed earlier and treated properly [12]. Herein, surgeons must have strongly vigilant for patients at risk of ACS preoperatively when faced with tibial plateau fractures, and taking preventive measures is much more important than the treatment of ACS after its occurrence.

Actually, much of the more recent published literatures have been made to focus on the prevalence rate and associated risk factors of ACS with tibial shaft fractures [13–15]. Shadgan et al. [15] previously concluded that ACS is more common among young people, which brings about an enormous socioeconomic burden should it occur. However, there is a relative paucity of literatures looking comprehensively at fracture patterns and ACS nor has there been few approaches to detect any relevant predictors to identify the possibility of ACS. It is generally accepted that higher-grade Schatzker classification and AO/OTA classification play a critical role in the development of ACS and correlate to the subsequent ACS occurrence closely [6, 7, 16, 17]. A recent retrospective cohort study reporting that the presence of a non-contiguous tibial fracture or knee dislocation was a radiographic predictor of ACS [18]. However, to the best of our knowledge, there have been few large series of studies on the prevalence rate of ACS and reported associated risk factors concomitant with tibial plateau fractures to date.

Therefore, the purpose of this study was to investigate the prevalence rate and predictors of the development of ACS following tibial plateau fractures. In particular, the results of this study may help orthopedic trauma surgeons to deepen understanding of the increased risk factors for the development of ACS, estimate the probability of ACS concomitant with tibial plateau fractures, and make a better decision when facing these concomitant complications.

## Patients and methods

**Fig. 1:**
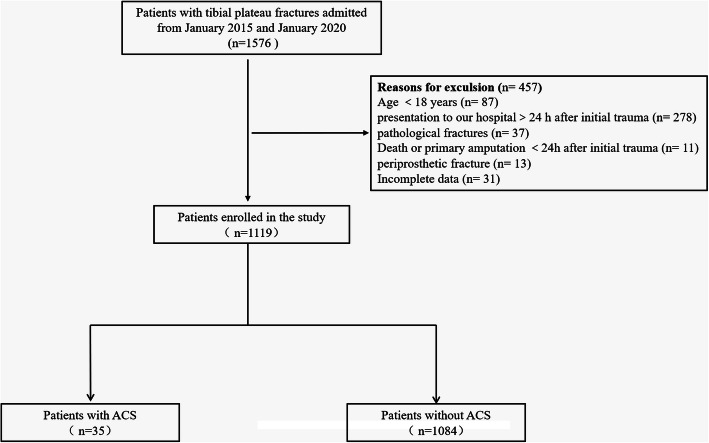
Flow diagram of patient enrollment

The study was conducted at a university-affiliated hospital with a level-I trauma center. A total of 1976 consecutive patients with tibial plateau fracture between January 2015 and January 2020 were retrospective included, of which 1519 patients were further analyzed according to the following exclusion criteria (Fig. 1). The exclusion criteria were as follows: (a) age younger than 18 years at the time of injury, (b) ipsilateral femoral or noncontiguous tibial shaft fracture, (c) admission to our hospital>24 h after the initial trauma, (d) pathological fracture (such as bone metastasis of tumor) or old fractures that more than 3 weeks, (e) periprosthetic fracture or intercondylar eminence fracture alone, (f) amputation or death<24 h after the initial trauma, and (g) incomplete medical records. This study was approved by the institutional ethical committee of our hospital and was conducted in accordance with the Declaration of Helsinki.

### Diagnosis of ACS

ACS was defined as occurring within the first 24 h after initial trauma in patients with tibial plateau fractures and diagnosed based on the combination of clinical symptoms and physical examination with available objective diagnostic data of testing [5, 19]. Furthermore, this definition was used widely in published studies [18, 20]. Diagnostic in clinical symptoms and signs associated with ACS included pain out of proportion to the injury, pain with passive stretch of the muscles, severe swelling, sensory impairment, or motor deficit [5, 21]. Intra-compartmental pressure (ICP) monitoring is one of the methods of objective diagnostic tool, which the pathological ICP values for ACS are an increase in pressure of more than 30 mm Hg for at least 2 h [22]. However, clinical diagnosis may be difficult to perform due to intubated, obtunded, or sedated patients after trauma, and ICP measurements were not performed routinely. The presence of color change in the muscle necrosis or muscle bulging at the time of fasciotomy is the gold standard for diagnosing ACS. Therefore, patients’ electronic medical records with operation reports were reviewed for confirming the “actual” number occurrence of ACS, as previously published study [13].

### Associated predictors in ACS following tibial plateau fractures

Demographics of a patient and injury-related factors associated with the development of ACS were collected from patients’ electronic medical records within the first 24 h after the initial trauma. Demographics of patients including age, gender, and underlying disease including hypertension, diabetes mellitus, and steroid use. Injury-related variables included mechanisms of injury, fracture pattern (open or closed), and fracture type based on the Schatzker classification. Mechanism of injury was classified as a slip from ground level, a fall from a height, motor vehicle collision, and others.

According to the Schatzker classification system, tibial plateau fractures were classified as follows: type I, split fracture of the lateral plateau without depression; type II, split, and depression of the lateral plateau; type III, pure depression fracture of the lateral plateau; type IV, split, or depressed fracture of the medial plateau; type V, bicondylar plateau fracture; and type VI, bilateral plateau fractures associated with metaphysis fracture.

### Statistical analysis

Univariate analysis was conducted for comparison of differences with continuous variables between patients with and without ACS using Mann-Whitney *U* test or Student’s *t* test, depending on the normality status of the variables. Chi-square tests or Fisher’s exact test was used to compare the difference for categorical variables. Multivariate logistical regression analysis was performed to investigate the independent predictors of ACS occurrence. SPSS software (version 25.0, IBM Corp., USA) was performed for statistical analysis.

## Results

From January 2015 to January 2020, 1576 patients with tibial plateau fractures were enrolled in this study. There were 457 patients who were excluded from our study protocol according to the exclusion criteria. Among these patients, 87 patients (4.4%) were younger than 18 years, 278 patients (14.1%) who presented to our hospital exceeded 24 h after the initial injury, 37 patients (1.9%) were pathological fractures, 11 patients (0.6%) were amputation or death within the first 24 h after the initial trauma, 13 patients (0.6%) were periprosthetic fractures, and 31 patients (1.6%) did not have complete medical records. Therefore, a total of 1119 patients including 703 males and 416 females with an average age of 40.7 years (18 to 80 years) were eligible for our study protocol. Of the 1119 tibial plateau fractures, 35 (3.1%) patients were associated with the development of ACS.

### Independent predictors for the development of ACS

**Fig. 2:**
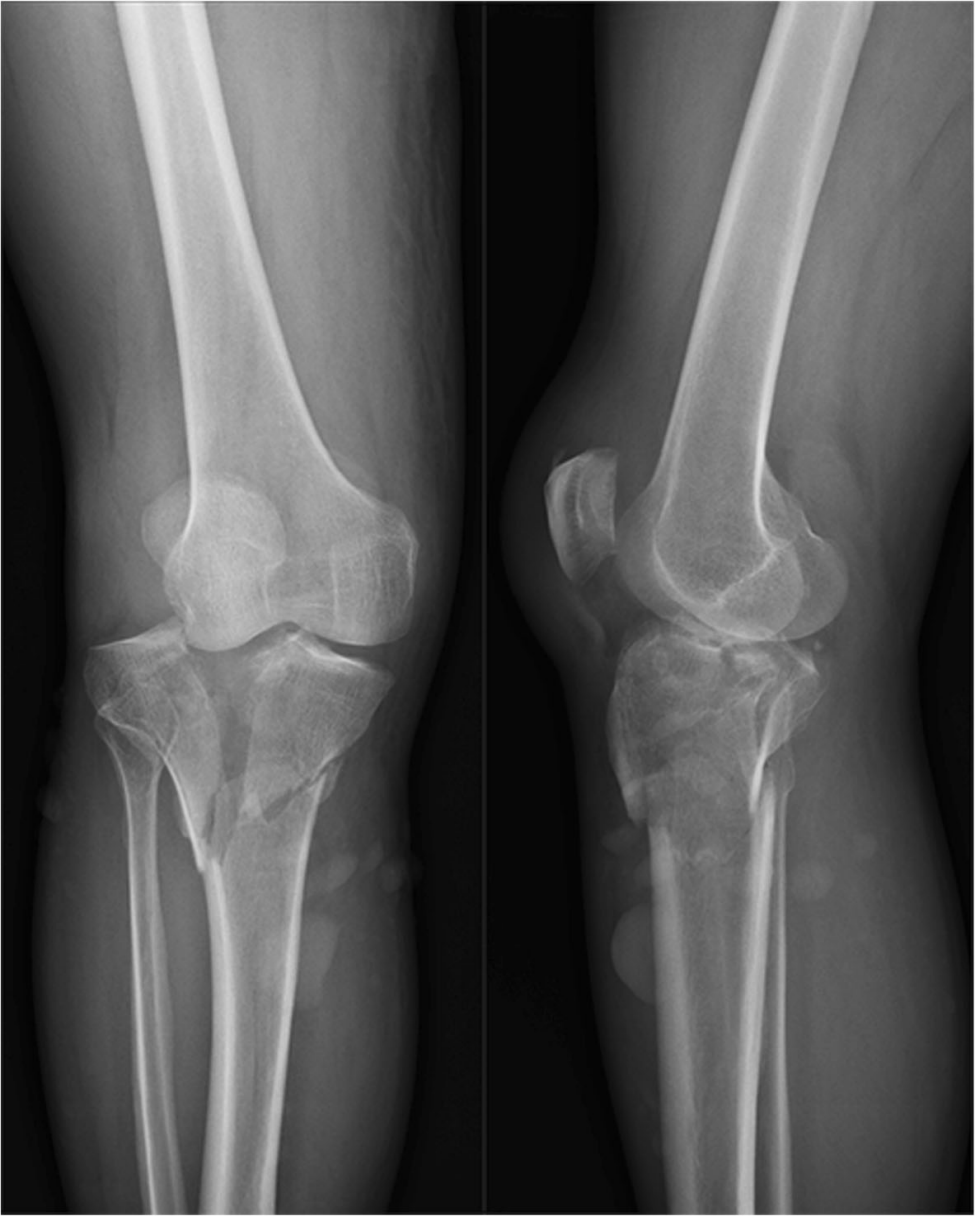
Radiographs of Schatzker type VI fracture, showing widening of the tibial plateau and comminution of the articular surface. The definition of a Schatzker type VI fracture is bilateral plateau fractures associated with metaphysis fracture

Table 1 shows the characteristics and injury mechanisms in patients with and without ACS after tibial plateau fractures. Univariate analysis identified that younger patient age and Schatzker type VI fracture (Fig. 2) were significantly associated with the development of ACS. Other factors, such as the mechanisms of injury, meantime from injury to admission, fracture pattern (open versus closed), and underlying disease (hypertension, diabetes mellitus, and steroid use), were not found to be significantly associated with the development of ACS (Table 1). In the multivariate logistic regression model, however, only younger patient age (odds ratio (OR) = 2.57; *P* = 0.003, logistic regression analysis) and Schatzker type VI fractures (odds ratio (OR) = 5.78; *P* = 0.021, logistic regression analysis) were statistically significant independent predictors for the development of ACS (Table 2).

**Table 1:** Univariate logistic regression analysis to identify predictors of acute compartment syndrome in the lower leg following tibial plateau fractures

Variable	with ACS (*n*=35)	Without ACS (*n*=1084)	*p*
Mean age, years	36.3±15.1	43.5±18.4	< 0.001*
Gender, *n* (%)			0.161
Female	9 (25.7)	407 (37.5)	
Male	26 (74.3)	677 (62.5)	
Mechanism of injury, *n* (%)			0.880
Motor vehicle accident	17 (48.6)	464 (42.9)	
Fall from a height	13 (37.1)	389 (36.0)	
Ground level fall	2 (5.7)	99 (9.1)	
Other	3 (8.6)	130 (12.0)	
Mean time from injury to admission, hours	2.1±1.3	2.7±3.4	0.674
Schatzker classification, (%)			
Type I	0 (0)	45 (4.2%)	N/A
Type II	0 (0)	346 (31.9%)	N/A
Type III	0 (0)	71 (6.5%)	N/A
Type IV	1 (2.9)	212 (19.6%)	0.856
Type V	7 (20.0)	169 (15.6%)	0.412
Type VI	27 (77.1)	241(22.2%)	0.017*
Fracture pattern, *n* (%)			0.758
Open	17 (48.6)	336 (31.0)	
Closed	18 (51.4)	748(69.0)	
Underlying disease, *n* (%)			0.854
Hypertension	7 (77.8)	292 (72.1)	
Diabetes mellitus	2 (22.2)	102 (25.2)	
Steroid use	0(0)	11 (2.7)	

^*^Statistically significant (*p*<0.05)

N/A, not applicable

**Table 2:** Results of multivariate logistic regression analysis using age and Schatzker type VI fracture for predictors in the development of ACS following tibial plateau fractures

Variables	OR	95%CI (lower limit)	95%CI (upper limit)	*P*
Age	2.57	1.26	6.31	0.003^†^
Schatzker type VI	5.78	1.78	54.34	0.021^†^

^†^Statistically significant (*p*<0.05)

## Discussion

In this study, we identified predictors of the development of ACS in the lower leg after a fracture of the tibial plateau, and our results had profound effects on the evaluation of these patients. Of note, our results demonstrated that the prevalence rate of ACS after tibial plateau fractures was 3.1% within the first 24 h after the initial trauma, which is lower than reported in previous literatures. Although previous studies were conducted to have identified the association between characteristics of clinical and radiographic of this specific fracture and the occurrence of ACS [18], this study shows those associations in a significantly large group of patients. Furthermore, multivariate logistical regression analysis found that only younger patient age and Schatzker type VI fractures were identified as independent predictors for ACS occurrence in adults with tibial plateau fractures. Our results demonstrated that there was no association between the mechanisms of injury and occurrence of ACS, which indicates that the amount of energy released to the soft tissue after initial trauma is a more important variable than the fracture-related injury mechanisms. Another explanation may be the injury mechanisms were not a reliable indicator of the energy of the initial trauma delivered to the soft tissue.

Age was proved to be a critical factor affecting the rate of ACS in our study, whereas multivariate analysis showed that male sex was not an independent predictor of ACS in tibial plateau fractures. Consistent with our findings, Park et al. [13] have found that decreasing age was an independent predictor for the development of compartment syndrome. Similarly, a study of adult patients showed that the lower leg of ACS is more frequent in patients younger than 45 years of age than those older than 45 years [18]. In the present research using both univariate and multivariate analysis, we identified that younger patient age was a significant predictor for the development of ACS. In the present research using both univariate and multivariate analysis, we identified that younger patient age had a higher prevalence rate of acute compartment syndrome than the older age, this could be explained by the fact that younger individuals had a relatively increased muscle mass, less compartment size, and thicker and less yielding fascial coverings due to a larger collagen content. This can cause the pressure to develop rapidly within a compartment with a small increase in volume. Furthermore, younger age people having larger muscle bulk within a tight fascia with little room to expand before the intra-compartmental pressure rises, which result in relatively higher prevalence rate in acute compartment syndrome. Turnipseed et al. [23] reported that the elevated intra-compartmental pressure is proportional to the stiffness of the fascia. Therefore, it has been supposed that there exist differences in the thickness and stiffness of the fascia between younger and older patients. We believed that this is the best explanation for the increased risk of ACS in younger patients. In addition, another reason may be attributed to the relatively higher number of high-energy trauma in younger patients, while the elderly often suffer from more low-energy fractures. Nevertheless, we still make a recommendation for older patients vigilant with compartment syndrome.

In prior literatures, approximately 10.4% of patients with tibial plateau fractures developed an acute compartment syndrome of the lower leg [18]. In our study, however, only 3.1% of patients developed an acute compartment syndrome. There were two possible explanations for our results. Firstly, there may have been some missing cases possibly because this study was retrospective. Furthermore, there is a lack of serial follow-up data of patients’ clinical outcome with a fracture of the tibial plateau, which may result in underestimating the prevalence rate of ACS. Secondly, the prevalence rate of ACS seems to have decreased among tibial plateau fractures due to the development of the healthcare system and improved safety equipment for medical care.

Interestingly, Blick et al. [24] previously demonstrated that the development of ACS is directly proportional to the degree of injury to soft tissue. In another study, Gamulin et al. [18] also observed that higher a linear association between the development of ACS and severity of soft tissue injury as well, which is in association with an increased amount of fascial and muscle injuries. Theoretically, open fractures always result from high-energy trauma and the severity of soft tissue injuries is a contributory factor in the occurrence of ACS; therefore, open fractures should be considered risk factors for the development of ACS. In contrast to prior literatures, however, the results of our study identified that there was no significant difference between closed fractures and open fractures regarding the prevalence rate of ACS in tibial plateau fractures. Literature on this topic is controversial, as some studies showed an association between open fractures and ACS occurrence [18, 25, 26] while others did not [13]. Indeed, we suggest that clinicians should recognize the soft tissue wounds around the fracture as a direct indicator of an increased amount of underlying ACS occurrence.

The amount of initial high-energy dissipated on the soft tissue during trauma, possibly causing the occurrence of ACS. Schatzker VI type fracture, involving bicondylar tibial plateau fractures associated with metaphyseal disruption, thus probably indicating a higher energy fracture pattern. In the present study, Schatzker VI type fracture was identified as an independent predictor for developing ACS of the lower extremity, which could be explained by the fact that Schatzer VI type could result in more energy transmitted to the soft tissue envelope (fascial and muscle injury) at the time of injury. Therefore, we concluded that the Schatzer VI type is an indicator of a high amount of energy delivered to the surrounding soft tissues, causing extensive soft tissue injuries potentially inducing the development of ACS. Of note, eight of our 35 patients with ACS had fracture types other than Schatzker type VI fracture. Although the prevalence rate of ACS is much higher than Schatzker type VI fracture, orthopedic trauma surgeons also need to be aware that other fracture patterns can result in ACS despite a lower prevalence rate.

However, this retrospective study has several inherent limitations that should be considered in future research. Firstly, the patients enrolled in our study were reviewed retrospectively in a single center based on patients’ electronic medical record, that is, our results depend on what was written in the medical record and its accuracy. Secondly, the relatively small number of patients with ACS may have a bias for analysis of our results. Thirdly, another limitation of our study is its retrospective design, thus limited data collection and influencing factors that were available included in the analysis. Future studies with large cohorts and prospective design would be helpful for including more influencing factors and further confirmation for predicting the risk factors of acute compartment syndrome. Last but not least, ICP measurements were not performed on all patients with tibial plateau fracture when admitting to our hospital, which indicated that there is a possibility of false-negative diagnosis (missed acute compartment syndrome). Therefore, studies with prospective measurement of compartment pressure and serial follow-up of patients with a fracture of the tibial plateau are still mandatory to focus on evaluating the prevalence rate of ACS. Despite these limitations of our study, this study is the largest series ever performed exploring associated predictors for the occurrence of ACS after tibial plateau fractures and has profound effects on the assessment of ACS.

## Conclusion

In conclusion, this study identified that younger patient age and Schatzker type VI fracture were predictors of acute compartment syndrome after tibial plateau fractures. Acute compartment syndrome remains a challenging condition and these consequences can be catastrophic, but long-term sequelae can be avoided by an earlier diagnosis of acute compartment syndrome and prompt emergency fasciotomy.

## Data Availability

All the data and material involving this article will be available upon request by sending an e-mail to the first author.

## Abbreviations

ACS: Acute compartment syndrome
